# Urdu translation and validation of the Urogenital Distress Inventory (UDI-6) in women with urinary incontinence

**DOI:** 10.1080/2090598X.2019.1618523

**Published:** 2019-06-03

**Authors:** Nuzhat Faruqui, Novera Chughtai, Jamil Ahmed

**Affiliations:** a Department of Urology, Aga Khan University, Karachi, Pakistan; b Department of Obstetrics and Gynaecology, Aga Khan University, Karachi, Pakistan

**Keywords:** Urdu, questionnaire translation, urogenital distress inventory (UDI), urinary incontinence, validation

## Abstract

**Objective**: To provide an Urdu translation of the six-item version of the Urogenital Distress Inventory (UDI-6) and its validation in patients with urinary incontinence (UI), as the UDI-6 is a recognised, useful disease-specific questionnaire for the evaluation of UI in women.

**Patients and methods**: We used a multi-step linguistic translation of the UDI-6, which comprised backward and forward translations coordinated by clinical investigators, followed by a pre-test in 10 patients. The final version was completed by a larger sample of women (*n* = 200), of which 100 had UI for the last 3 months and 100 had no UI. To appraise test–retest reliability the patients with UI were re-tested after 2 weeks. To test the questionnaire’s capacity to discriminate between women with or without UI, both cases (patients) and controls were included and assessed. The reliability of the UDI-6 was evaluated by internal consistency and was calculated using the Wilcoxon signed-rank test with *P* values, and test–retest reliability assessed by Spearman’s coefficient with *P* values.

**Results**: The reliability of the UDI-6 was assessed for internal consistency and test–retest reliability was evaluated by Spearman’s coefficient, which showed significant *P* values.

**Conclusion**: The present Urdu version of the UDI-6 is a linguistically valid instrument that can be reliably used in clinical practice and research.

**Abbreviations:** IIQ-7: seven-item version of the Incontinence Impact Questionnaire; IQR: interquartile ranges; QoL: quality of life; UDI-6: six-item version of the urogenital distress inventory; UI: urinary incontinence

## Introduction

According to the recommendations of the ICS/International Urogynecological Association (IUGA) joint report 2010, urinary incontinence (UI) is defined as ‘complaint of involuntary loss of urine’ [1].

The global prevalence of UI is ~49% for stress UI, 22% for urge UI, and 29% for mixed UI. UI is a very common disorder affecting females of all ages [2]. In a study from Pakistan involving 180 female patients, 44% of females reported significant UI, of which 17.2% and 16.1% complained of stress or mixed UI, respectively, with 9.7% complaining of urge UI only [3].

Various generic and disease-specific quality-of-life (QoL) questionnaires, such as the six-item version of the Urogenital Distress Inventory (UDI-6) and seven-item version of the Incontinence Impact Questionnaire (IIQ-7), are used to evaluate women presenting with UI in the West. Generic questionnaires use non-specific questions and are lengthy and complexity restricted. Therefore, it has been postulated that the simultaneous use of generic and disease-specific questionnaires will improve the sensitivity to assess the impact of UI on the QoL of women [4]. This sensitivity is increased if the questionnaire used is translated into the population’s native language, as confirmed by various studies, and therefore validated questionnaires, like the UDI-6 and IIQ-7, have been translated into various languages including: Arabic, Dutch and Turkish [5–8].

The national language of Pakistan is Urdu and many Pakistanis’ are unfamiliar with even basic English language. There is no validated QoL instrument in the Urdu language that can measure the impact of UI. Therefore, the aim of our present study was to provide an Urdu version of the UDI-6 questionnaire and validate its use in an Urdu-speaking population. As UI is considered as a social taboo, a questionnaire validated in the patient’s own language will help them report their issues accurately and comfortably, and also provide a platform for future research.

The objective of the present study was to provide an Urdu translation of the UDI-6 and its validation in female patients with UI.

## Patients and methods

### Study design

A cross-sectional study was conducted at the outpatient urology clinic over a 4-month period. Patients (cases) were asked to complete the questionnaire at baseline and 2 weeks later, so as to evaluate the test–retest reliability. The controls were assessed only once, as in patients with LUTS their symptoms would be seen again for clinical reasons, whilst in controls they would not. Therefore, the test–retest reliability was performed on patients only. During the study period, the questionnaires were completed by the patients as well as the controls. However, the translation process itself took 6–7 months prior to use. The UDI includes 18 items; thus, a sample of 180 patients would be ideally required, so we included 200. The sample size of 200 was adequate to fulfill the above criteria and objective measures of urinary symptoms.

The inclusion criteria of the study were all female patients aged ≥18 years reporting UI for the last 3 months. These were the cases. The controls were all healthy subjects with no UI for the last 3 months. The exclusion criteria included all those whose education status was below primary level, those with psychiatric diseases, and those with incomplete forms. We also excluded those patients with language barriers. These were patients from distant places or from other countries who did not understand Urdu or English, and needed to have a translator present for history taking and to follow various instructions during examination.

### Translation process

The validation process of any measure in a new language depends on a rigorous linguistic translation, which permits conceptual and technical equivalence between the original source and target language as much as possible [9]. The following steps were performed for the linguistic validation:

1. Translation of the UDI-6 from the original language (English) to Urdu was done by two independent Urdu-speaking translators with English as their first foreign language (Appendix S1).
2. The first consensus meeting included the two translators and the research group, who compared the Urdu versions, and developed a first Urdu version of the UDI-6.
3. A back-translation of this Urdu version was done by a native English-speaking translator, with Urdu as their first foreign language.
4. The second consensus meeting was held between the English translator and investigators, during which the back-translation and the original UDI-6 were compared, and the first version was revised.
5. A pilot test was done, to assess whether the UDI-6 was clear to the target population, in 10 patients (i.e., women with urological problems) [10]. The number of patients who had problems in correctly understanding the questions and pre-coded answers were recorded and discussed.
6. The final Urdu version of the UDI-6 was devised.

The questionnaire was first introduced at the patient’s initial visit to the clinic and then at the first follow-up visit, which usually was 2 weeks later. Here the questionnaire was re-introduced to the patients and they completed it. The total number of participants was 200, of which 100 were cases and 100 were controls.

### Statistical analysis

The analysis was done using the Statistical Package for the Social Sciences (SPSS®), version 20 (SPSS Inc., IBM Corp., Armonk, NY, USA). The sample size for this study was 200 and the recommended subject-to-item ratio of a given measurement scale should be 5:1 or above. The reliability of the UDI-6 was assessed by internal consistency and was calculated using the Wilcoxon signed-rank test with *P* values, and test–retest reliability was evaluated by Spearman’s coefficient with *P* values.

### Ethical considerations

Ethics approval for the study was granted by the local institute.

## Results

In the control group of 100 females the mean (SD) age was 30 (8) years. In the cases group of 100 patients, the mean (SD) age was 46 (16) years.

Table 1 shows that the questionnaire was able to discriminate primarily between the cases and controls, by calculating medians with interquartile ranges (IQRs).

**Table 1.** Comparison of median score (IQR) using Mann–Whitney *U*-test between cases and control at baseline.

	Median (IQR) score		
UDI-6 symptoms	Cases	Controls	P*
Frequency	2 (1–3)	0 (0–1)	<0.001
Urgency	2 (1–3)	0 (0–1)	<0.001
Stress UI	2 (1–3)	0 (0–1)	<0.001
UI volume	2 (1–3)	0 (0–1)	<0.001
Difficulty urinating	2 (1–3)	0 (0–1)	<0.001
Pain	1 (1–3)	0 (0–1)	<0.001

The correlation between the baseline and 2-week symptoms score was calculated in cases using Spearman’s correlation coefficient. These are the values of each individual item at first visit (baseline) and at 2 weeks, as shown in scatter plots. This is done to check the test–retest reliability, i.e., to see if each item of the questionnaire, which the cases and controls have answered is the same after 2 weeks.

Table 2 shows the correlation between the baseline and 2-week symptoms score in cases, which were calculated using Spearman’s correlation coefficient. All of them show significant *P* values, which means that when looking at the test–retest reliability, all the items were reliable and they can be used in a real sense.

**Table 2.** Correlation between symptom severity at baselines and 2 weeks.

UDI-6 symptom	Spearman’s ρ	*P*
Frequency	0.845	<0.001
Urgency	0.901	<0.001
Stress UI	0.887	<0.001
UI volume	0.855	<0.001
Difficulty urinating	0.993	<0.001
Pain baseline	1	<0.001

Internal consistency was calculated using Wilcoxon signed-rank test (Table 3). The significant *P* values indicate that the correlation between the severities of the symptoms was valid and consistent.

**Table 3.** Consistency between baseline and 2-week self-reported symptom severity amongst cases (*n* = 54).

	Symptom severity			
UDI-6 symptom	Decreased	Increased	Unchanged	*P*
Frequency	6	3	45	0.454
Urgency	2	3	49	0.334
Stress UI	4	3	47	0.792
UI volume	4	2	48	0.666
Difficulty urinating	1	0	53	0.317
Pain baseline	0	0	54	1.00

* Wilcoxon signed-ranks test was used to assess consistency between baseline and 2-week self-reported symptom severity amongst cases.

## Discussion

The UDI-6 is a condition-specific QoL questionnaire, which is simple to understand and provides a more in-depth assessment of specific concerns related to UI [6]. However, its validation in the Chinese language proves that there may be cultural discrepancies and language-specific issues, e.g., items may need to be adjusted according to cultural differences in different populations [11]. We also plan to assess the need for modification according to our cultural needs in a later study.

In the present study, there was a high correlation between ratings, meaning that the questionnaire is stable for short time-intervals. This is similar to various studies where this questionnaire has been validated in national languages, the validation in Chinese showed a Pearson’s coefficient value of 0.8 [11]. In contrast, the linguistic validation in Swedish language showed an α coefficient of 0.39 [12].

Generally, internal consistency is considered ‘good’ when the α coefficient is >0.7. The internal consistency was 0.76 for the original version by Wyman et al. [13] and it was satisfactory in an Italian version by Artibani et al. [14]. In our present study, the *P* values of all variables were significant, thus proving good internal consistency.

The present results show that the Urdu translation of the UDI-6 is a reliable and valid instrument. All forward and backward translations were consistent with each other and the original English version. We followed the standard translation method, consisting of forward and backward translations, and a consensus meeting between the clinical investigators and the translators. This process is a widely accepted methodology for linguistic validation [10].

## Conclusion

The present Urdu version of the UDI-6 is a valid instrument that can be reliably used in daily practice and clinical research.

## Supplementary material

Translated UDI-6
