# Supplementary figures and images for: Urdu translation and validation of the Urogenital Distress Inventory (UDI-6) in women with urinary incontinence
Source: Arab J Urol. 2019 Jun 3;17(3):212–5. doi: 10.1080/2090598X.2019.1618523 (PMC6711080; doi:10.1080/2090598X.2019.1618523)

# Appendix S1. Translated UDI-6.


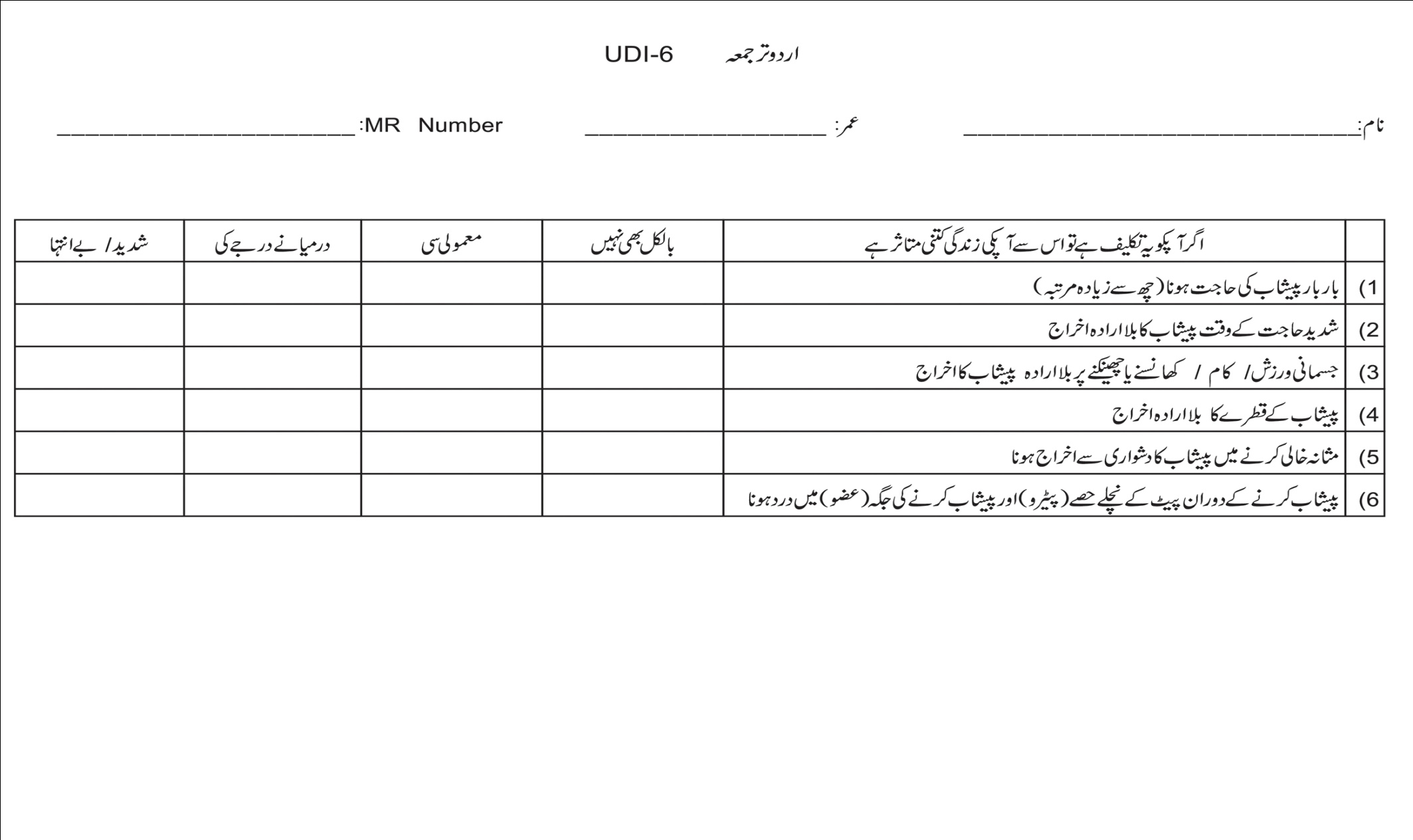

Supplement: Translated UDI-6 [file TAJU_A_1618523_SM2030.doc]
